# Anti-osteoporotic effects of mixed compositions of extracellular polymers isolated from *Aureobasidium pullulans* and *Textoria morbifera* in ovariectomized mice

**DOI:** 10.1186/s12906-018-2362-y

**Published:** 2018-11-06

**Authors:** Chang-Soo Cho, Hye-Seong Jeong, In-Young Kim, Go-Woon Jung, Bon-Hwa Ku, Dong-Chan Park, Seung-Bae Moon, Hyung-Rae Cho, Khawaja Muhammad Imran Bashir, Sae Kwang Ku, Jae-Suk Choi

**Affiliations:** 1 Hurim Hwangchil Co., Ltd, 907-11, Gyeongseo-dearo, Hoengcheon-myeon, Hadong-gun, Gyeongsangnam-do 52320 Republic of Korea; 2 Glucan Corp., #305 Marine Bio-Industry Development Centger, 7 Hoenggye-gil, Ilgwang-myeon Busan, 46048 Republic of Korea; 3 Research Center for Extremophiles and Microbiology, College of Medical and Life Sciences, Silla University, 140, Baegyang-daero 700 beon-gil, Sasang-gu Busan, 46958 Republic of Korea; 4 Seafood Research Center, IACF, Silla University, 606, Advanced Seafood Processing Complex, Wonyang-ro, Amnam-dong, Seo-gu Busan, 49277 Republic of Korea; 5 Department of Anatomy and Histology, College of Korean Medicine, Daegu Haany University, 1, Hanuidae-ro, Gyeongsan-si, Gyeongsangbuk-do 38610 Republic of Korea; 6 Major in Food Biotechnology, Division of Bioindustry, College of Medical and Life Sciences, Silla University, 140, Baegyang-daero 700beon-gil, Sasang-gu Busan, 46958 Republic of Korea

**Keywords:** Anti-osteoporotic effect, *Aureobasidium pullulans*, β-Glucans, Exopolymer, *Textoria morbifera*

## Abstract

**Background:**

Extracellular polymeric substances isolated from *Aureobasidium pullulans* (EAP), containing specifically 13% β-1,3/1,6-glucan, have shown various favorable bone-preserving effects. *Textoria morbifera* Nakai (TM) tree has been used as an ingredient in traditional medicine and tea for various pharmacological purposes. Thus, the present study was aimed to examine the synergistic anti-osteoporotic potential of mixtures containing different proportions of EAP and TM compared with that of the single formulations of each herbal extract using bilateral ovariectomized (OVX) mice, a renowned rodent model for studying human osteoporosis.

**Methods:**

Thirty five days after bilateral-OVX surgery, 9 combinations of EAP:TM (ratios = 1:1, 1:3, 1:5, 1:7, 1:9, 3:1, 5:1, 7:1, 9:1) and single separate formulations of EAP or TM were supplied orally, once a day for 35 days at a final concentration of 200 mg/kg. Variations in body weight gains during the experimental periods, as well as femur weights, bone mineral density (BMD), bone strength (failure load), and mineral content (calcium [Ca] and inorganic phosphorus [IP]) following sacrifice were measured. Furthermore, histomorphometric and histological profile analyses of serum biochemical parameters (osteocalcin content and bone specific alkaline phosphatase [bALP] activity) were conducted following sacrifice. Femurs histomorphometric analyses were also conducted for bone resorption, structure and mass. The results for the mixed formulations of EAP:TM and separate formulations were compared with those of risedronate sodium (RES).

**Results:**

The EAP:TM (3:1) formulation synergistically enhanced the anti-osteoporotic potential of individual EAP or TM formulations, possibly due to enhanced variety of the active ingredients. Furthermore, the effects of EAP:TM were comparable to those of RES (2.5 mg/kg) treatment.

**Conclusion:**

The results of this study suggest that, the EAP:TM (3:1) combination might act as a new pharmaceutical agent and/or health functional food substance for curing osteoporosis in menopausal women.

**Electronic supplementary material:**

The online version of this article (10.1186/s12906-018-2362-y) contains supplementary material, which is available to authorized users.

## Background

Osteoporosis is becoming an increasingly critical public health problem owing to the aging population. Although it is partially preventable, fractures related to osteoporosis are still common [1]. Osteoporosis, a metabolic bone disorder, causes an imbalance between the bone [2]. Thus, osteoporosis constitutes a major public health issue due to its association with age-related fractures, particularly of the hip, vertebrae, distal forearm, and humerus [3, 4]. During the past decade, numerous studies have been reported on the effects of new substances for the prevention and/or treatment of bone disorders [5, 6]. However, there is a need to develop an efficient resorptive inhibitor with higher safety. On-going trials to develop anabolic agents are typically designed through understanding bone formation and the differences in osteoblast [7–9].

Osteoclast-mediated bone resorption is inhibited by binding of risedronate sodium (RES) to bone hydroxyapatites [10]. RES, a pridinyl bisphosphonate, inhibits bone resorption by changing osteoclast cytoskeleton protein [11], and induces osteoclast apoptosis [12]. RES has been previously used for the curing osteoporosis in menopausal women [13]. Topical treatments with RES (at a concentration of 0.02% and 2.5 mg/kg/day) in OVX rat and/or mice have shown sustained bone biomechanical and microstructural features [14, 15]. Hence, after OVX, oral administration of risedronate sodium (2.5 mg/kg per day) was employed as a positive-control.

*Textoria morbifera* (TM) Nakai mainly grows in Jeju-do Island and along the Korean southwestern coastline [16, 17]. It has been used as an ingredient for traditional medicine and tea for immunological enhancement [16–18]. Previously, TM extracts have exhibited numerous immunological activities, including anti-diabetic [19], antioxidant [20], anti-cancer [21], anti-insecticidal [22], anti-complementary effects, as well as antibiotic and medicinal effects for liver diseases [23]. Recently, TM extracts have been reported for anti-osteoclastogenic and osteoporosis ameliorating effects [24–26]. β-selinene, a sesquiterpene derivative and capnellane-8-one have been reported as the most abundant active ingredients of TM [27].

Polysaccharides are linked with host defense mechanisms [28]. The immunopharmacological effects of exopolymers, β-1,3/1,6-glucans, have been reported by in vitro*,* in vivo and clinical studies as well [29]. The key immunological features include anti-tumor activity [30], radioprotective actions [31], increased host resistance to microbial infections [32], as well as adjuvant effects [33]. In addition, exopolysaccharides isolated from *Aureobasidium pullulans* (EAP) contain 13% β-1,3/1,6-glucan [34, 35] as a major ingredient. The exopolymers isolated from *A. pullulans* have demonstrated favorable anti-osteoporotic [8, 35], fracture healing [36], anti-inflammatory [37, 38], and potent immunomodulatory activities in mouse models [39]. These extracts have also shown favorable nephroprotective effects [40], ameliorative activity against ovalbumin-induced asthma [41], and anti-osteoarthritic effects [42, 43].

It has been reported that the various pharmacological effects of natural products are synergistically increased by appropriate mixed formulations [9, 44–46]. Specifically, the bone-preserving characteristics of EAP have been potentiated by combining it with other active agents [47–49]. Therefore, it was hypothesized that the mixed formulations of exopolymer (EAP) and leaf extracts of TM (EAP:TM) might show favorable synergistic anti-osteoporotic properties due to enhanced availability of the bioactive substances.

In this study, we aimed to investigate the optimal compositions of EAP:TM mixtures that are associated with clear synergistic anti-osteoporotic potential compared with the single formulations, using bilateral OVX female mice, a renowned rodent model for studying human osteoporosis [7, 9].

## Methods

### Animal husbandry

**Fig. 1:**
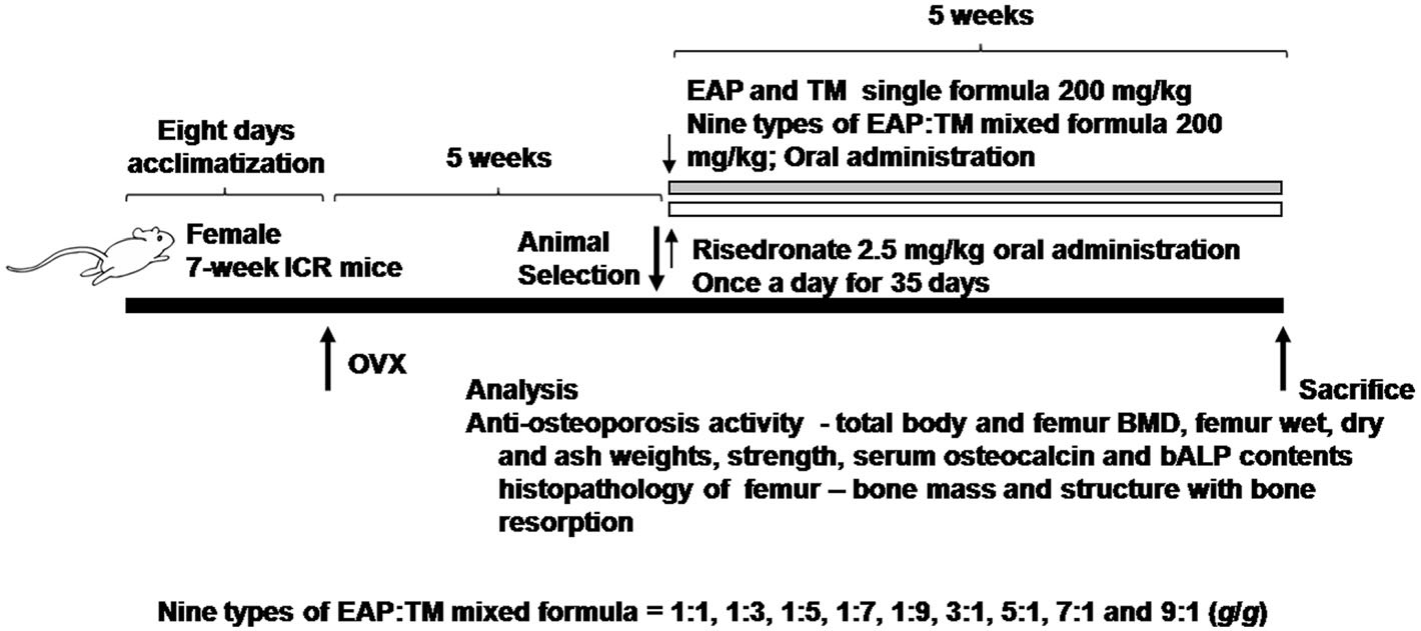
Study experimental design. OVX: Bilateral ovariectomy; RES: Risedronate sodium; EAP: Exopolymers purified from *A. pullulans* SM2001; TM: *T. morbifera* leaf extracts; (EAP:TM): Mixed formula consisted of EAP and TM (*g*/*g*)

One hundred and sixty virgin female SPF/VAF (CrljOri:CD1 [ICR]; 7-weeks old) mice, purchased from OrientBio, Seungnam, Republic of Korea, were acclimatized for 8 days and then used for these experiments. Four mice were assigned per polycarbonate cage and reared in a humidity (45–55%), and temperature (20–25 °C), −controlled room. Light was provided for 12 h and the standard rodent chow diet (Purinafeed, Seungnam, Rep. of Korea) and water were provided freely. In this experiment, 150 mice were allocated as OVX-operated osteoporosis model and 10 mice were allocated as sham-operated control. After 34 days of OVX operation, 8 mice from each group were chosen depending on body weight deviations (OVX-mice: 37.38 ± 2.71 g, range of 34.2 ~ 44.4 g; sham-operated mice: 33.86 ± 2.53 g, range of 30.3 ~ 37.5 g). The experimental designs for this study are presented in Fig. 1, and the animals were assigned to the experimental groups as follows:

1. Sham vehicle control: Sham-operated and distilled water administered
2. OVX control: OVX and distilled water administered
3. RES: OVX and treated with RES (2.5 mg/kg)
4. EAP: OVX and treated with 200 mg/kg of EAP single formulation
5. TM: OVX and treated with 200 mg/kg of TM single formulation
6. OVX mice treated with 9 different EAP:TM mixed formulations
  6-1. EAP:TM 1:1 (g/g) 200 mg/kg (100:100 mg/kg)
  6-2. EAP:TM 1:3 (g/g) 200 mg/kg (50:150 mg/kg)
  6-3. EAP:TM 1:5 (g/g) 200 mg/kg (33:167 mg/kg)
  6-4. EAP:TM 1:7 (g/g) 200 mg/kg (25:175 mg/kg)
  6-5. EAP:TM 1:9 (g/g) 200 mg/kg (20:180 mg/kg)
  6-6. EAP:TM 3:1 (g/g) 200 mg/kg (150:50 mg/kg)
  6-7. EAP:TM 5:1 (g/g) 200 mg/kg (167:33 mg/kg)
  6-8. EAP:TM 7:1 (g/g) 200 mg/kg (175:25 mg/kg)
  6-9. EAP:TM 9:1 (g/g) 200 mg/kg (180:20 mg/kg)

### Preparation and administration of test materials

EAP, an extracellular polymer isolated from *A. pullulans* (SM-2001; marketed as Polycan™) and the standard leaf extract of *T. morbifera* (TM) were obtained from Glucan Corp., Busan, Rep. of Korea. EAP consisted of 40% total β-glucans including 13% β-1,3/1,6-glucans [34, 35]. EAP and TM were mixed thoroughly in distilled water to a final concentration of 20 mg/ml. A few samples of TM [Code No: TM2016Ku01], and EAP [Code No: EAP2016Ku01] were deposited in the herbarium, Medical Research Center for Globalization of Herbal Formulation, Daegu Haany University, Gyeongsangbuk-do, Gyeongsan-si, Rep. of Korea. The 9 different herbal formulations at a final concentration of 200 mg/kg/body weight (EAP:TM = 1:1, 1:3, 1:5, 1:7, 1:9, 3:1, 5:1, 7:1, and 9:1) and both single formulations of TM and EAP were supplied orally, once a day for 35 days after 35 days of OVX surgery. The EAP:TM mixed formulations (1:1, 1:3, 1:5, 1:7, 1:9, 3:1, 5:1, 7:1, and 9:1) were prepared by dissolving EAP and TM (100:100, 50:150, 33:167, 25:175, 20:180, 150:50, 167:33, 175:25, and 180:20 mg:mg) in distilled water, respectively (final concentration: 200 mg in 10 ml of distilled water). The prepared mixtures were then provided at a concentration of 200 mg/kg, by gastric gavages using a 1 ml syringe. The single formulations of EAP and TM were also prepared and administrated in the same manner as other mixed formulations of EAP and TM.

In addition, 0.25 mg risedronate sodium (RES; TEVA Tapi, Sheva, Israel) was also dissolved in 1 ml distilled water and provided orally at a concentration of 2.5 mg/kg [10, 15]. The control groups (OVX and Sham control) were provided with equal volumes of distilled water (vehicle) instead of test substance to provide the same experimental conditions and administration stress.

### Osteoporosis induction

A mixture of 2 to 3% isoflurane [2–3% isoflurane in 28.5% O_2_ and 70% N_2_O], was used to anesthetize the animals using a rodent inhalation and rodent ventilator system and the animals were maintained with 1 to 1.5% isoflurane. The surgical protocol was carried out according to the previously reported methods [7–9]. In the OVX treatment group, bilateral OVX was removed by open surgery and the incisions were stitched in two steps. The bilateral OVX surgery was not performed in the sham operated mice group (2nd group). The animals were sacrificed by cervical dislocation under anesthesia using 99.50% CO_2_ gas and the stomach was excised with a median incision in the abdomen.

### Body weight measurements

The changes in body weights were recorded on weekly basis by an automatic electronic balance. At the initiation and termination of sample administration as well as on the day of OVX, all trial mice were starved for approximately 18 h (only water was supplied) to decrease alterations due to feeding. Furthermore, body weight gains were estimated by Eq. [1], and Eq. [2].

1 $$ \mathrm{Body}\ \mathrm{weight}\ \mathrm{gains}\ \mathrm{after}\ \mathrm{administration}\ \left(35\ \mathrm{days}\right)=\mathrm{Body}\ \mathrm{weight}\ \mathrm{at}\ \mathrm{sacrifice}-\mathrm{Body}\ \mathrm{weight}\ \mathrm{at}\ \mathrm{initial}\ \mathrm{provision}\ \mathrm{of}\ \mathrm{test}\ \mathrm{material} $$

2 $$ \mathrm{Body}\ \mathrm{weight}\ \mathrm{gains}\ \mathrm{during}\ \mathrm{OVX}\ \mathrm{recovery}/\mathrm{induced}\ \mathrm{periods}\ \left(35\ \mathrm{days}\right)=\mathrm{Body}\ \mathrm{weight}\ \mathrm{at}\ \mathrm{initial}\ \mathrm{provision}\ \mathrm{of}\ \mathrm{test}\ \mathrm{material}-\mathrm{body}\ \mathrm{weight}\ \mathrm{at}\ \mathrm{OVX}\ \mathrm{surgery} $$

### Estimation of BMD

In vivo dual-energy X-ray absorptiometry was used to estimate the changes in mean BMD of the right femur and the total body, 24 h after the final administration of the test materials.

### Measurement of bone weight

After 35 days continuous treatment with the test material, right side of the femur was collected and the bone absolute wet-weights were recorded after weighting the bones; however, dry-weights were measured after drying the bones at 200 °C for 8 h. In addition, the bone absolute ash weights were measured after carbonizing the dried bones in a furnace at 800 °C for 6 h [8]. The individual body weight variances were minimized by calculating the relative weights (% body weights) using the Eq. [3].

3 $$ \mathrm{Relative}\ \mathrm{bone}\ \mathrm{weight}\mathrm{s}=\left[\left(\mathrm{Absolute}\ \mathrm{bone}\ \mathrm{weight}/\mathrm{Body}\ \mathrm{weight}\ \mathrm{at}\ \mathrm{s}\mathrm{acrifice}\right)\times 100\right] $$

### Estimation of bone strength

Bone strengths [Failure load (FL); unit: Newton (N)] of the mid-shaft regions of femurs (dried right femur) were measured through a three-point bending failure stress test by a computerized testing machine as reported previously [8, 50].

### Serum biochemical analysis

At sacrifice, whole blood (approximately 1 ml) was collected in a serum collection tube and the serum was separated by centrifugation at 15,000 rpm for 10 min under 4 °C. All collected samples were stored at − 150 °C using an Ultra-deep freezer (Sanyo, Tokyo, Japan) until further analysis. Mouse Osteocalcin ELIZA Kit and Mouse bALP ELIZA Kit, purchased from MyBioSource (CA, USA), were used for measuring the serum osteocalcin and bALP levels, respectively.

### Femur mineral contents

The ground bone ash powder was dissolved in nitric acid (HNO_3_) and the inorganic phosphate (IP) and calcium (Ca) contents were measured by the previously reported *o*-cresolphthalein complexone [7], and enzyme [8] methods, respectively. Furthermore, Ca/IP ratios were determined by Eq. [4].

4 $$ \mathrm{Bone}\ \mathrm{Ca}/\mathrm{IP}\ \mathrm{ratio}=\left[\left(\mathrm{Bone}\ \mathrm{Ca}\ \mathrm{content}/\mathrm{Bone}\ \mathrm{IP}\ \mathrm{content}\right)\right]\times 100 $$

### Histopathological analyses of bone

The separated left femur of each mouse was fixed in 10% neutral buffered formalin and decalcified for 3 days in a decalcifying solution [24.4% formic acid and 0.5 N sodium hydroxide]. Following decalcification, femur trochlea head regions were longitudinally trimmed, embedded in paraffin, sectioned in 3–4 μm, and stained with hematoxylin and eosin (HE). In each prepared histological sample, the histological profiles were interpreted. The prepared histological samples were coded for blind histopathological analysis. Bone histomorphometry was performed by an automated image analyzer and the bone structure, resorption, and mass were measured in the cortical or epiphyseal regions of the femur. Thickness of the cortical bone was measured in the femurs’ mid-shaft region. Trabecular bone thickness (Tbt), length (Tbl), number (Tbn) and volume (TBV), and thickness of cortical bone (Cbt) for structure and mass, and osteoclast cell number (Ocn) and osteoclast cell ratios (OS/BS) were estimated for bone resorption [7–9].

### Statistical analysis

The experimental data is stated as mean ± standard deviation (S.D.) of eight animals. Different doses of test material were compared by the multiple comparison tests. Levene’s test was used to examine the variance homogeneity [51]. If no significant deviation was observed by the Levene’s test, then the obtained data was evaluated by one-way ANOVA by least-significant differences multi-comparison (LSD) test. If a significant deviation from variance was observed by the Levene’s test, groups were compared by a non-parametric comparison test (Kruskal-Wallis H test). If the Kruskal-Wallis H test showed a significant difference, the Mann-Whitney U test was performed to estimate the significantly different specific pairs of groups. Differences were considered significant at *p < 0.05* and/or *p < 0.01*. SPSS ver. 14 (IBM-SPSS Inc., Chicago, IL, USA) was used for the statistical analyses [52]. To estimate the effectiveness of the test material, the percent fluctuations between treated-mice and the OVX-control were estimated respectively by Eq. [5], and Eq. [6] as demonstrated previously [53].

5 $$ \mathrm{Percent}\ \mathrm{differences}\ \mathrm{compared}\ \mathrm{with}\ \mathrm{OVX}\ \mathrm{control}\ \left(\%\right)=\left[\left\{\left(\mathrm{data}\ \mathrm{for}\ \mathrm{test}\ \mathrm{material}\ \mathrm{treated}\ \mathrm{mice}-\mathrm{data}\ \mathrm{for}\ \mathrm{OVX}\ \mathrm{control}\right)/\mathrm{data}\ \mathrm{for}\ \mathrm{OVX}\ \mathrm{control}\right\}\times 100\right] $$

6 $$ \mathrm{Percent}\ \mathrm{differences}\ \mathrm{compared}\ \mathrm{with}\ \mathrm{the}\ \mathrm{sham}\ \mathrm{control}\ \left(\%\right)=\left[\left\{\left(\mathrm{data}\ \mathrm{for}\ \mathrm{OVX}\ \mathrm{control}\ \mathrm{mice}-\mathrm{data}\ \mathrm{for}\ \mathrm{sham}\ \mathrm{control}\ \mathrm{mice}\right)/\mathrm{data}\ \mathrm{for}\ \mathrm{sham}\ \mathrm{control}\ \mathrm{mice}\right\}\times 100\right] $$

## Results

### Changes in body weight and weight gain

Eight mice from each group showing significant (*p < 0.05*) increases in body weights were selected for OVX models 34-days post-surgery [OVX mice: 37.38 ± 2.71 g, weight range of 34.2 g to 44.4 g; sham-operated mice: 33.86 ± 2.53 g, weight range 30.3 g to 37.5 g]. All OVX-control groups revealed significant increases in body weights. Furthermore, significantly (*p < 0.01*; *p < 0.05*) higher body weight gains were observed in OVX-mice during the 5-weeks of OVX recovery/induction period. Although, obvious decreases in body weights were noticed during some periods of treatment with EAP:TM 1:1, 1:5, 3:1, and 7:1 as compared with the OVX-mice; animals treated with all EAP and TM formulations showed significant (*p < 0.01*) decreases in body weight gains. Specifically, EAP:TM (3:1) treated OVX mice revealed significant (*p < 0.05*) reductions in body weight gains as compared with mice treated with other formulations. RES (2.5 mg/kg)-treated mice showed no significant variations in body weights and body weight gains throughout the entire experimental period (Table 1; Additional file 1).

**Table 1:** Body weight gain in sham-operated and OVX mice

Periods Groups	Body weights (g)				Body weight gains (g)	
	At OVX [A]*	At 34 days after OVX [B]	At initial treatment [C]*	At sacrifice [D]*	OVX recovery [B-A]	Treatment [D-C]
Controls						
Sham	25.55 ± 1.62	33.86 ± 2.53	29.90 ± 2.56	31.45 ± 2.74	8.31 ± 1.88	1.46 ± 0.92
OVX	25.51 ± 1.20	37.40 ± 3.22^b^	33.96 ± 2.84^a^	37.59 ± 2.75^a^	11.89 ± 2.90^a^	3.63 ± 0.73^a^
Reference (2.5 mg/kg)						
RES	26.29 ± 0.80	36.68 ± 1.71^b^	33.40 ± 1.68^b^	35.75 ± 2.80^a^	10.39 ± 1.53^b^	2.35 ± 2.11
Single formula (200 mg/kg)						
EAP	26.61 ± 1.59	37.70 ± 3.22^a^	34.00 ± 2.62^a^	35.08 ± 1.90^a^	11.09 ± 3.11^b^	1.08 ± 1.76^c^
TM	25.99 ± 1.35	37.31 ± 3.20^b^	34.10 ± 2.99^a^	35.06 ± 2.57^a^	11.33 ± 2.55 ^a^	0.96 ± 0.78^c^
Mixed formula - EAP:TM (200 mg/kg)						
1:1	25.60 ± 1.82	36.59 ± 1.87	33.18 ± 1.83^b^	34.53 ± 2.11^bd^	10.99 ± 2.10^a^	1.35 ± 1.47^c^
1:3	26.80 ± 2.21	37.63 ± 3.03^a^	34.26 ± 2.74^a^	35.24 ± 3.58^a^	10.83 ± 2.29^b^	0.98 ± 1.41^c^
1:5	25.60 ± 1.01	37.58 ± 2.92^b^	33.80 ± 2.63^a^	34.69 ± 3.91^bd^	11.98 ± 3.34^a^	0.89 ± 2.03^c^
1:7	26.73 ± 1.88	37.49 ± 2.95^b^	33.84 ± 2.53^a^	34.91 ± 2.49^b^	10.79 ± 2.27^b^	1.08 ± 2.23^c^
1:9	26.39 ± 1.14	37.55 ± 2.98^b^	34.08 ± 2.67^a^	35.23 ± 1.75^a^	11.16 ± 2.53^a^	1.15 ± 1.73^c^
3:1	25.93 ± 1.27	37.51 ± 2.99^b^	33.79 ± 2.76^a^	32.76 ± 2.66^c^	11.59 ± 2.83^a^	- 1.03 ± 1.43^acef^
5:1	26.13 ± 1.24	37.54 ± 2.87^b^	34.00 ± 2.75^a^	34.99 ± 2.52^b^	11.41 ± 3.27^a^	0.99 ± 1.92^c^
7:1	26.11 ± 0.96	37.48 ± 2.89^b^	33.96 ± 2.69^a^	34.91 ± 3.10^b^	11.36 ± 2.62^a^	0.95 ± 1.25^c^
9:1	26.44 ± 1.66	37.50 ± 2.80^b^	34.05 ± 2.79^a^	35.05 ± 2.15^a^	11.06 ± 2.50^a^	1.00 ± 1.71^c^

*OVX* Bilateral ovariectomy, *RES* Risedronate sodium, *EAP* Exopolymers purified from *A. pullulans* SM2001, *TM T. morbifera* leaf extracts, *(EAP:TM)* Mixed formula consisted of EAP and TM (*g*/*g*)

Values are expressed mean ± S.D. of eight mice

*All animals were overnight fasted

^a^*p* < 0.01 and ^b^*p* < 0.05 as compared with sham control by LSD test

^c^*p* < 0.01 and ^d^*p* < 0.05 as compared with OVX control by LSD test

^e^*p* < 0.05 as compared with EAP single formula treated mice by LSD test

^f^*p* < 0.05 as compared with TM single formula treated mice by LSD test

### Effects on femur weight

OVX control groups showed significant decreases in absolute and relative wet, dry, and ash weights of femur as compared with that in sham control groups. Conversely, noticeable increases in wet, dry, and ash weights of femur were demonstrated by all mice administered with test substances, including RES as compared with OVX controls. In particular, EAP:TM 3:1-treated groups exhibited significantly (*p < 0.01*; *p < 0.05*) higher femur relative wet-weights, and absolute and relative dry and ash weights as compared with the mice administered with EAP or TM single formulations (Table 2; Additional file 2).

**Table 2:** Right femur weights of sham-operated and OVX mice

Items Groups	Absolute weight (g)			Relative weight (% of body weight)		
	Wet	Dry	Ash	Wet	Dry	Ash
Controls						
Sham	0.094 ± 0.006	0.067 ± 0.004	0.049 ± 0.003	0.300 ± 0.030	0.214 ± 0.016	0.157 ± 0.012
OVX	0.091 ± 0.006	0.047 ± 0.003^a^	0.027 ± 0.002^a^	0.243 ± 0.017^a^	0.126 ± 0.012^a^	0.071 ± 0.007^a^
RES 2.5 mg/kg	0.098 ± 0.007	0.066 ± 0.004^c^	0.046 ± 0.003^bc^	0.274 ± 0.022^bc^	0.185 ± 0.019^ac^	0.128 ± 0.012^ac^
EAP 200 mg/kg	0.093 ± 0.006	0.058 ± 0.005^ac^	0.034 ± 0.003^ac^	0.266 ± 0.008^ad^	0.164 ± 0.007^ac^	0.097 ± 0.005^ac^
TM 200 mg/kg	0.094 ± 0.007	0.058 ± 0.005^ac^	0.035 ± 0.003^ac^	0.269 ± 0.026^bd^	0.166 ± 0.013^ac^	0.100 ± 0.008^ac^
Mixed formula - EAP:TM (200 mg/kg)						
1:1	0.094 ± 0.007	0.056 ± 0.005^ac^	0.035 ± 0.003^ac^	0.272 ± 0.029^bd^	0.163 ± 0.019^ac^	0.102 ± 0.011^ac^
1:3	0.095 ± 0.009	0.058 ± 0.005^ac^	0.035 ± 0.004^ac^	0.270 ± 0.024^bd^	0.165 ± 0.015^ac^	0.100 ± 0.012^ac^
1:5	0.093 ± 0.007	0.057 ± 0.004^ac^	0.034 ± 0.003^ac^	0.270 ± 0.026^bd^	0.166 ± 0.025^ac^	0.100 ± 0.014^ac^
1:7	0.094 ± 0.004	0.056 ± 0.002^ac^	0.034 ± 0.001^ac^	0.272 ± 0.027^bd^	0.162 ± 0.015^ac^	0.098 ± 0.009^ac^
1:9	0.096 ± 0.008	0.058 ± 0.005^ac^	0.035 ± 0.002^ac^	0.272 ± 0.025^bd^	0.165 ± 0.018^ac^	0.100 ± 0.009^ac^
3:1	0.098 ± 0.009	0.065 ± 0.003^cef^	0.044 ± 0.004^cef^	0.300 ± 0.027^ceg^	0.200 ± 0.017^cef^	0.134 ± 0.011^acef^
5:1	0.095 ± 0.008	0.056 ± 0.005^ac^	0.036 ± 0.004^ac^	0.272 ± 0.021^bd^	0.169 ± 0.014^ac^	0.103 ± 0.016^ac^
7:1	0.094 ± 0.009	0.058 ± 0.005^ac^	0.035 ± 0.003^ac^	0.268 ± 0.017^ad^	0.166 ± 0.010^ac^	0.100 ± 0.006^ac^
9:1	0.095 ± 0.005	0.060 ± 0.005^ac^	0.035 ± 0.003^ac^	0.271 ± 0.022^bd^	0.171 ± 0.012^ac^	0.100 ± 0.007^ac^

*OVX* Bilateral ovariectomy, *RES* Risedronate sodium, *EAP* Exopolymers purified from *A. pullulans* SM2001, *TM T. morbifera* leaf extracts, *(EAP:TM)* Mixed formula consisted of EAP and TM (*g*/*g*)

Values are expressed mean ± S.D. of eight mice

^a^*p* < 0.01 and ^b^*p* < 0.05 as compared with sham control by LSD test

^e^*p* < 0.01 as compared with EAP single formula treated mice by LSD test

^c^*p* < 0.01 and ^d^*p* < 0.05 as compared with OVX control by LSD test

^f^*p* < 0.01 and ^g^*p* < 0.05 as compared with TM single formula treated mice by LSD test

### Effects on serum biochemical parameters: Osteocalcin content and bALP activities

**Fig. 2:**
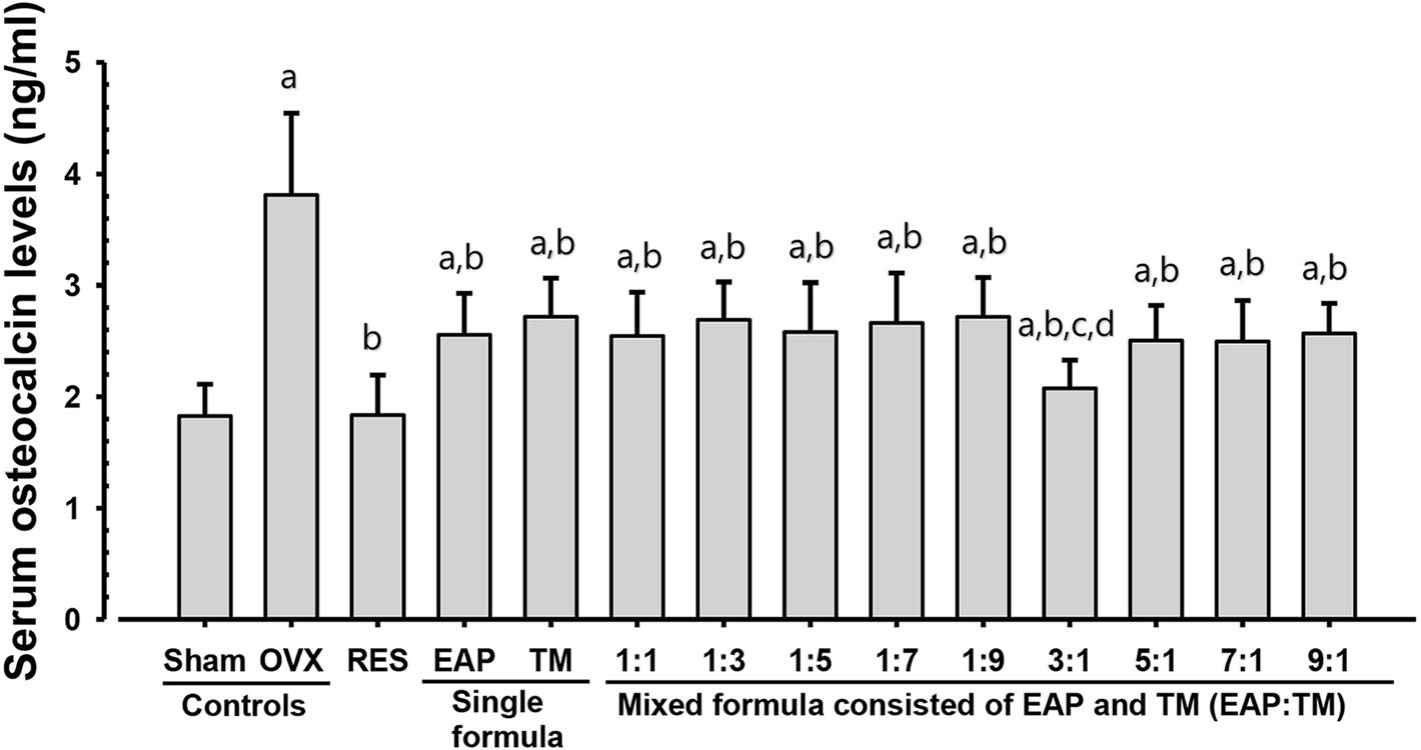
Serum osteocalcin levels in sham-operated and OVX mice. OVX: Bilateral ovariectomy; RES: Risedronate sodium; EAP: Exopolymers purified from *A. pullulans* SM2001; TM: *T. morbifera* leaf extracts; (EAP:TM): Mixed formula consisted of EAP and TM (*g*/*g*). Values are expressed mean ± S.D. of eight mice. RES was orally administered at a dose level of 2.5 mg/kg. All single and mixed formula consisted of EAP and TM were administered at a dose level of totalized 200 mg/kg by gastric gavage. ^a^*p* < 0.01 as compared with sham control by LSD test. ^b^*p* < 0.01 as compared with OVX control by LSD test. ^c^*p* < 0.05 as compared with EAP single formula treated mice by LSD test. ^d^*p* < 0.01 as compared with TM single formula treated mice by LSD test

**Fig. 3:**
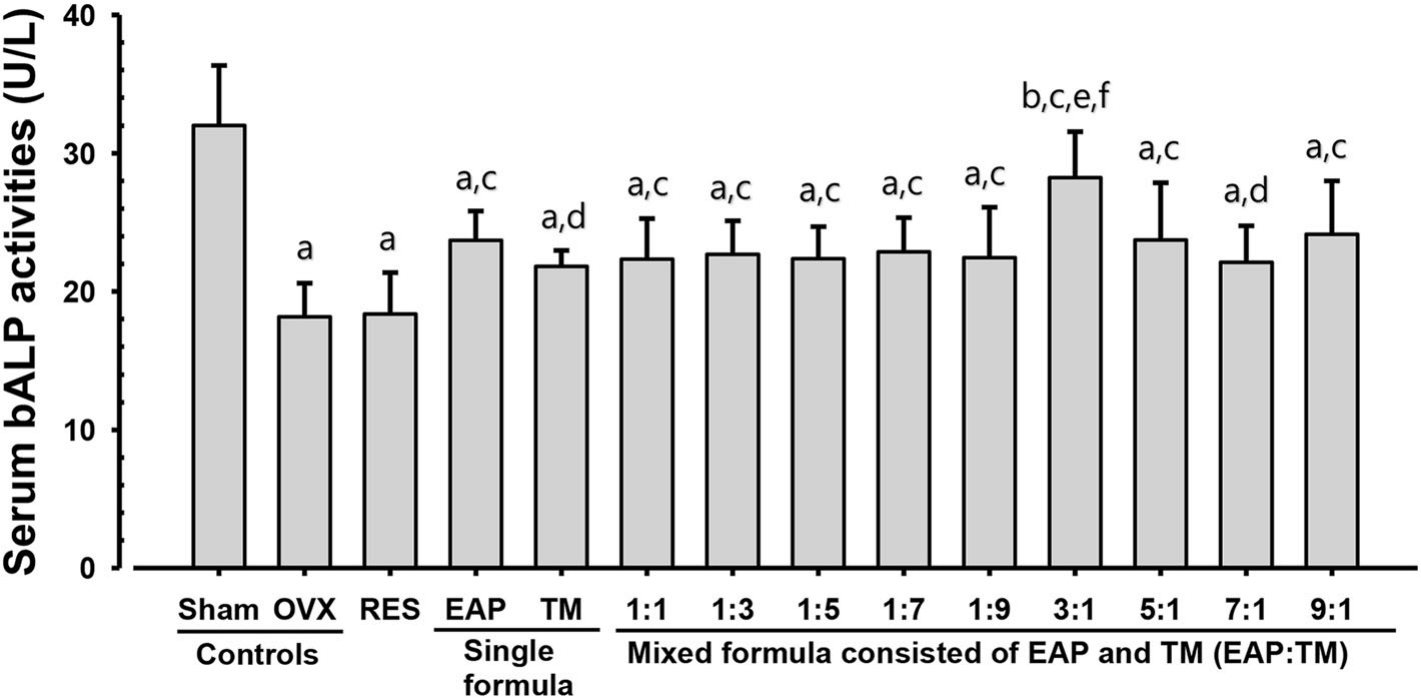
Serum bALP levels in sham-operated and OVX mice. OVX: Bilateral ovariectomy; RES: Risedronate sodium; EAP: Exopolymers purified from *A. pullulans* SM2001; TM: *T. morbifera* leaf extracts; (EAP:TM): Mixed formula consisted of EAP and TM (*g*/*g*). Values are expressed mean ± S.D. of eight mice. OVX = Bilateral ovariectomy; RES = Risedronate sodium; RES was orally administered at a dose level of 2.5 mg/kg. All single and mixed formula consisted of EAP and TM were administered at a dose level of totalized 200 mg/kg by gastric gavage. ^a^*p* < 0.01 and ^b^*p* < 0.05 as compared with sham control by LSD test. ^c^*p* < 0.01 and ^d^*p* < 0.05 as compared with OVX control by LSD test. ^e^*p* < 0.01 as compared with EAP single formula treated mice by LSD test. ^f^*p* < 0.01 as compared with TM single formula treated mice by LSD test

OVX control groups displayed a significant (*p < 0.01*) decline in serum bALP activity and a noteworthy rise in serum osteocalcin. However, all formulations of EAP and TM significantly (*p < 0.01*) reduced the serum osteocalcin levels and significantly (*p < 0.01*) increased the bALP activity. Particularly, EAP:TM (3:1) treated mice revealed significant (*p < 0.01; p < 0.05*) decreases in serum osteocalcin content and increases in serum bALP activity. RES (2.5 mg/kg) supplied OVX mice also showed significantly (*p < 0.01*) decreases in serum osteocalcin levels; yet, both groups demonstrated similar serum bALP activity compared to the OVX control group (Figs. 2 and 3; Additional file 3).

### Effects on femur BMD

**Fig. 4:**
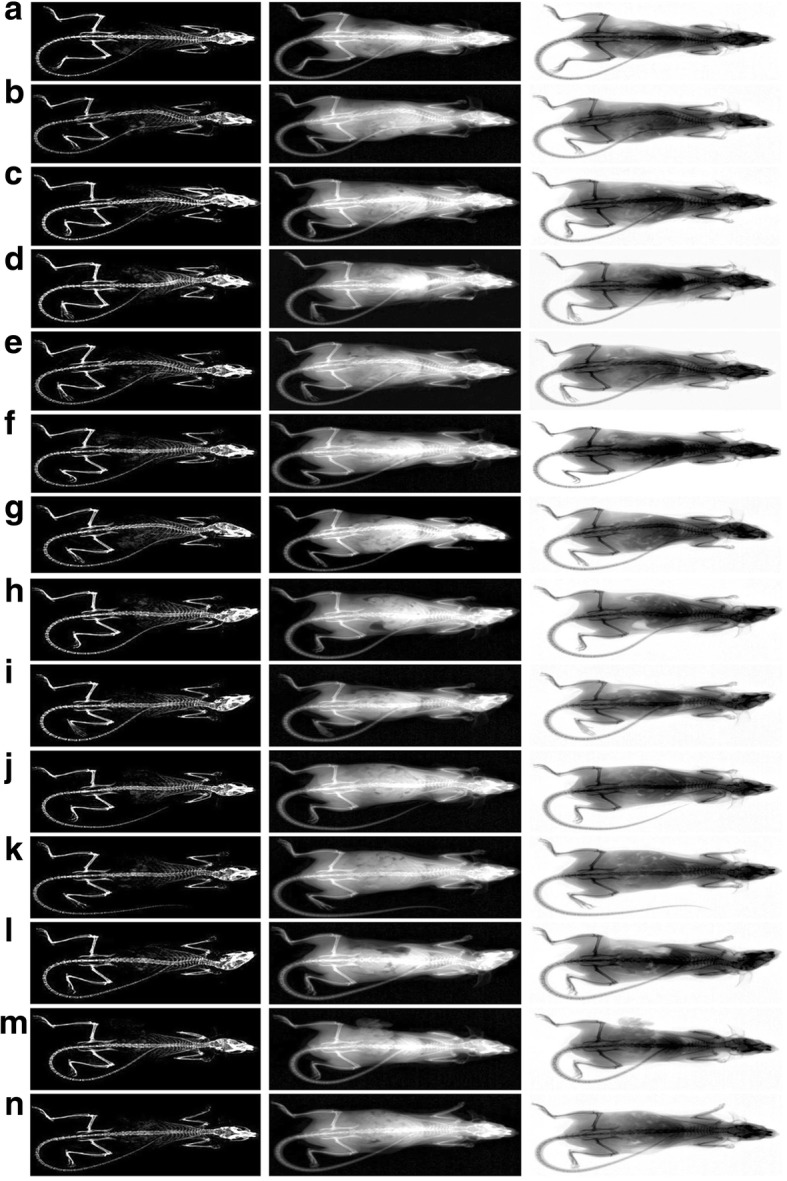
Representative whole body DEXA images, taken from sham-operated and OVX mice. **a** = Sham-operated and distilled water administered sham vehicle control mice. **b** = Distilled water administered OVX control mice. **c** = RES 2.5 mg/kg orally administered OVX mice. **d** = EAP single formula 200 mg/kg orally administered OVX mice. **e** = TM single formula 200 mg/kg orally administered OVX mice. **f** = EAP:TM 1:1 (g/g) mixed formula 200 mg/kg orally administered OVX mice. **g** = EAP:TM 1:3 (g/g) mixed formula 200 mg/kg orally administered OVX mice. **h** = EAP:TM 1:5 (g/g) mixed formula 200 mg/kg orally administered OVX mice. **i** = EAP:TM 1:7 (g/g) mixed formula 200 mg/kg orally administered OVX mice. **j** = EAP:TM 1:9 (g/g) mixed formula 200 mg/kg orally administered OVX mice. **k** = EAP:TM 3:1 (g/g) mixed formula 200 mg/kg orally administered OVX mice. **l** = EAP:TM 5:1 (g/g) mixed formula 200 mg/kg orally administered OVX mice. **m** = EAP:TM 7:1 (g/g) mixed formula 200 mg/kg orally administered OVX mice. **n** = EAP:TM 9:1 (g/g) mixed formula 200 mg/kg orally administered OVX mice. OVX: Bilateral ovariectomy; RES: Risedronate sodium; EAP: Exopolymers purified from *A. pullulans* SM2001; TM: *T. morbifera* leaf extracts; (EAP:TM): Mixed formula consisted of EAP and TM (*g*/*g*). DEXA: Dual-energy x-ray absorptionmetry

A significantly decreased total body and femur mean BMD were observed in OVX mice. However, the test substances-administered groups showed significantly increased femur mean BMD and total body, including the EAP single formulation, compared with that in OVX control mice. In particular, EAP:TM (3:1)-treated mice showed significant (*p < 0.01*) increased femur mean BMD and total body as compared to mice supplied with the single formulations of EAP or TM (Table 3; Fig. 4; Additional file 4).

**Table 3:** Bone femur strength, and mineral content (Ca and IP) in sham-operated and OVX mice

Items Groups	Bone Mineral Density (g/cm^2^)		Strength (*Newton*)	Right femur bone mineral contents		
	Total body	Right femur	Right femur	Ca (mg/g bone)	IP (mg/g bone)	Ca/IP ratio
Controls						
Sham	0.0249 ± 0.0007	0.0274 ± 0.0008	12.47 ± 1.66	59.08 ± 10.25	45.42 ± 7.57	1.30 ± 0.07
OVX	0.0215 ± 0.0003^f^	0.0231 ± 0.0001^a^	6.69 ± 0.91^f^	33.37 ± 3.32^f^	26.14 ± 3.26^f^	1.28 ± 0.11
RES 2.5 mg/kg	0.0251 ± 0.0008^h^	0.0262 ± 0.0013^bc^	11.27 ± 1.78^h^	55.33 ± 11.04^h^	44.03 ± 10.17^h^	1.26 ± 0.05
EAP 200 mg/kg	0.0233 ± 0.0003^fh^	0.0252 ± 0.0007^ac^	8.59 ± 0.63^fh^	42.76 ± 4.14^fh^	33.27 ± 3.18^fh^	1.29 ± 0.06
TM 200 mg/kg	0.0231 ± 0.0004^fh^	0.0248 ± 0.0006^ac^	7.96 ± 0.69^fi^	39.28 ± 3.84^fi^	31.32 ± 3.51^fh^	1.26 ± 0.06
Mixed formula - EAP:TM (200 mg/kg)						
1:1	0.0333 ± 0.0003^fh^	0.0253 ± 0.0006^ac^	8.32 ± 0.98^fh^	40.63 ± 2.92^fh^	31.79 ± 2.93^fh^	1.28 ± 0.07
1:3	0.0232 ± 0.0003^fh^	0.0253 ± 0.0008^ac^	8.04 ± 0.67^fi^	40.77 ± 3.43^fi^	32.39 ± 3.40^fi^	1.26 ± 0.06
1:5	0.0231 ± 0.0003^fh^	0.0250 ± 0.0010^ac^	8.23 ± 0.86^fh^	41.48 ± 4.10^fh^	32.85 ± 3.64^fh^	1.27 ± 0.09
1:7	0.0232 ± 0.0004^fh^	0.0252 ± 0.0009^ac^	8.08 ± 0.88^fh^	40.39 ± 1.58^fh^	31.51 ± 1.58^fh^	1.28 ± 0.04
1:9	0.0232 ± 0.0003^fh^	0.0249 ± 0.0008^ac^	8.29 ± 0.84^fi^	40.35 ± 4.00^fi^	32.17 ± 3.02^fi^	1.25 ± 0.05
3:1	0.0244 ± 0.0005^hjl^	0.0268 ± 0.0011^cde^	10.58 ± 1.17^ghjl^	48.96 ± 2.87^hhjl^	38.25 ± 2.96^ghkl^	1.28 ± 0.06
5:1	0.0234 ± 0.0006^fh^	0.0256 ± 0.0015^ac^	8.48 ± 0.81^fh^	42.67 ± 5.16^fh^	33.66 ± 4.52^fh^	1.27 ± 0.06
7:1	0.0232 ± 0.0004^fh^	0.0249 ± 0.0010^ac^	8.36 ± 0.54^fh^	42.25 ± 3.11^fh^	33.26 ± 3.28^fh^	1.28 ± 0.09
9:1	0.0232 ± 0.0003^fh^	0.0250 ± 0.0005^ac^	8.45 ± 0.69^fh^	40.21 ± 3.74^fh^	31.61 ± 2.00^fh^	1.27 ± 0.05

*OVX* Bilateral ovariectomy, *RES* Risedronate sodium, *EAP* Exopolymers purified from *A. pullulans* SM2001, *TM T. morbifera* leaf extracts, *(EAP:TM)* Mixed formula consisted of EAP and TM (*g*/*g*), *Ca* Calcium, *IP* Inorganic phosphorus

Values are expressed mean ± S.D. of eight mice

^a^*p* < 0.01 and ^b^*p* < 0.05 as compared with sham control by LSD test

^d^*p* < 0.01 as compared with EAP single formula treated mice by LSD test

^c^*p* < 0.01 as compared with OVX control by LSD test

^e^*p* < 0.01 as compared with TM single formula treated mice by LSD test

^f^*p* < 0.01 and ^g^*p* < 0.05 as compared with sham control by MW test

^j^*p* < 0.01 and ^k^*p* < 0.05 as compared with EAP single formula treated mice by MW test

^h^*p* < 0.01 and ^i^*p* < 0.05 as compared with OVX control by MW test

^l^*p* < 0.01 as compared with TM single formula treated mice by MW test

### Effects on bone strength

The strength (FL) of the mid-shaft region of the right dry femur in OVX mice showed noteworthy decreases; while, significant (*p < 0.01; p < 0.05*) increases in the femur FL were detected in all groups treated with test substances, including TM single formulations, compared with that in OVX controls. Specifically, EAP:TM (3:1)-treated mice consistently showed significant (*p < 0.01*) increases in the femur FL compared to mice treated with single formulations of EAP or TM (Table 3; Additional file 5).

### Changes in femur mineral contents

OVX mice showed significant (*p < 0.01*) decreases in femur Ca and IP contents. While, a significantly (*p < 0.01; p < 0.05*) increased trend in femur Ca and IP contents was observed in all groups treated with the test substances, especially in mice treated with EAP:TM (3:1). No noteworthy variation in femur Ca/IP ratio was observed in OVX mice compared with that in sham control. Furthermore, no significant differences in femur Ca/IP ratios were detected in mice administered with the test substances, including RES (2.5 mg/kg), compared with that in the OVX control group (Table 3; Additional file 6).

### Changes in femur histopathology

**Fig. 5:**
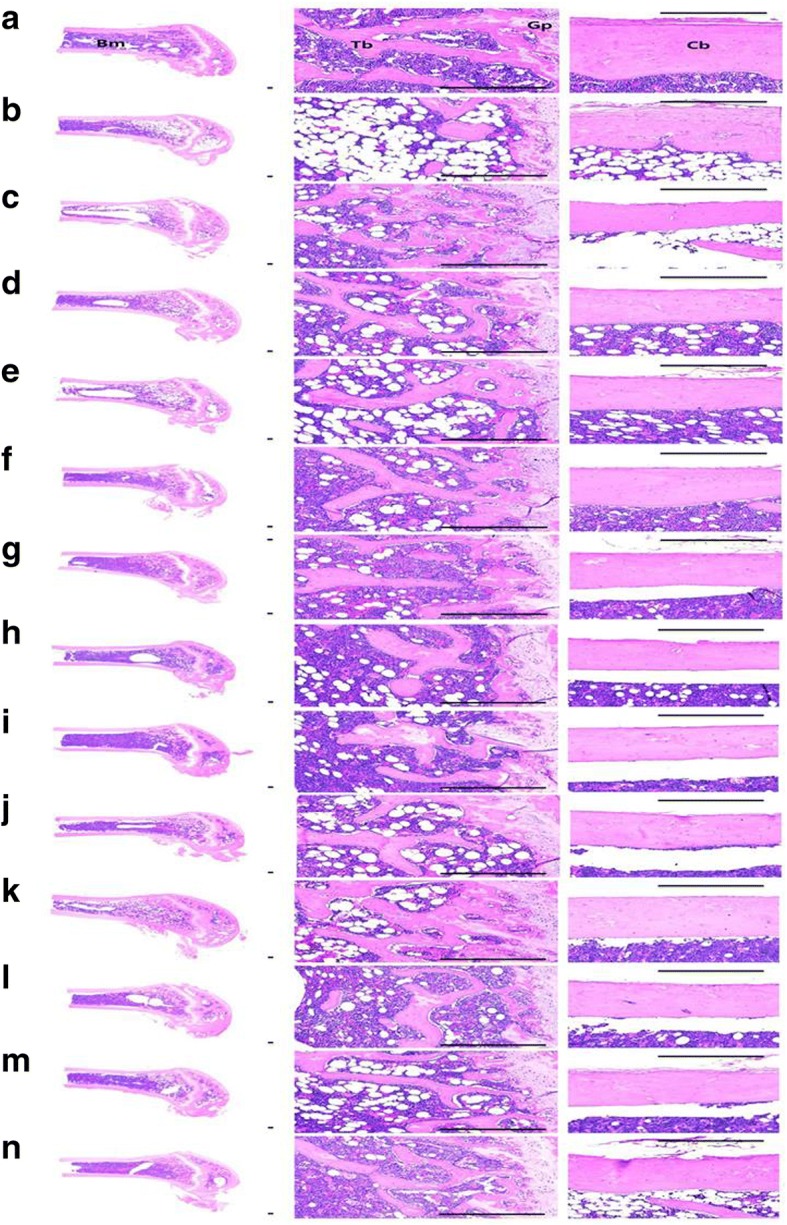
Representative histological profiles of the left femur, taken from sham-operated and OVX mice. **a** = Sham-operated and distilled water administered sham vehicle control mice. **b** = Distilled water administered OVX control mice. **c** = RES 2.5 mg/kg orally administered OVX mice. **d** = EAP single formula 200 mg/kg orally administered OVX mice. **e** = TM single formula 200 mg/kg orally administered OVX mice. **f** = EAP:TM 1:1 (g/g) mixed formula 200 mg/kg orally administered OVX mice. **g** = EAP:TM 1:3 (g/g) mixed formula 200 mg/kg orally administered OVX mice. **h** = EAP:TM 1:5 (g/g) mixed formula 200 mg/kg orally administered OVX mice. **i** = EAP:TM 1:7 (g/g) mixed formula 200 mg/kg orally administered OVX mice. **j** = EAP:TM 1:9 (g/g) mixed formula 200 mg/kg orally administered OVX mice. **k** = EAP:TM 3:1 (g/g) mixed formula 200 mg/kg orally administered OVX mice. **l** = EAP:TM 5:1 (g/g) mixed formula 200 mg/kg orally administered OVX mice. **m** = EAP:TM 7:1 (g/g) mixed formula 200 mg/kg orally administered OVX mice. **n** = EAP:TM 9:1 (g/g) mixed formula 200 mg/kg orally administered OVX mice. OVX: Bilateral ovariectomy; RES: Risedronate sodium; EAP: Exopolymers purified from *A. pullulans* SM2001; TM: *T. morbifera* leaf extracts; (EAP:TM): Mixed formula consisted of EAP and TM (*g*/*g*). Cb: cortical bone; Tb: trabecular bone; Bm: bone marrow; Gp: growth plate. All Hematoxylin and eosin stain. Scale bars = 300 μm

OVX control mice showed a typical osteoporotic histological profile, whereas left femur of the sham-control mice showed relatively well-developed cortical and trabecular bones. The periosteum of cortical bones showed increased connective tissues, however, a dramatic decrease in cortical and trabecular bone mass was observed. Although, RES reduced the trabecular bone loss, no changes in cortical bone masses were observed. However, a dramatic increase in bone structure and mass of cortical and trabecular bones was observed for all the tested formulations; especially, EAP:TM (3:1) treated groups showed a visible inhibition in the bone mass loss of femur and the histopathological activation of osteoclast cells than the other treatment groups (Tables 4 and 5; Fig. 5).

**Table 4:** Histopathology and histomorphometry of the femur in sham-operated and OVX mice: Trabecular Bones

Items Groups	Left femur tissues			
	TV/BV	Tbn	Tbl	Tbt
Controls				
Sham	39.33 ± 5.16	18.63 ± 2.56	967.87 ± 131.16	68.48 ± 10.39
OVX	11.33 ± 2.10^e^	3.38 ± 1.19^e^	318.15 ± 76.18^a^	35.91 ± 10.13^e^
Reference (2.5 mg/kg)				
RES	43.99 ± 7.02^g^	27.50 ± 5.53^eg^	828.28 ± 109.33^ab^	36.51 ± 10.81^e^
Single formula (200 mg/kg)				
EAP	24.75 ± 2.46^eg^	10.50 ± 1.60^eg^	594.88 ± 94.79^ab^	49.42 ± 2.60^eg^
TM	22.77 ± 3.11^eg^	8.75 ± 2.96^eg^	656.09 ± 127.21^ab^	48.86 ± 3.98^eg^
Mixed formula - EAP:TM (200 mg/kg)				
1:1	24.38 ± 3.73^eg^	9.88 ± 2.23^eg^	551.50 ± 64.08^ab^	48.70 ± 5.40^fh^
1:3	23.68 ± 3.09^eg^	10.25 ± 1.49^eg^	647.64 ± 147.56^ab^	49.76 ± 7.89^eh^
1:5	22.41 ± 3.53^eg^	9.63 ± 1.85^eg^	635.86 ± 99.62^ab^	52.72 ± 10.21^eg^
1:7	23.39 ± 3.11^eg^	9.38 ± 1.92^eg^	646.56 ± 62.11^ab^	49.70 ± 5.97^eg^
1:9	23.66 ± 3.97^eg^	10.88 ± 2.03^eg^	623.38 ± 86.57^ab^	52.38 ± 8.86^eg^
3:1	35.42 ± 4.00^gij^	17.88 ± 1.96^gij^	913.57 ± 151.07^bcd^	61.78 ± 6.26^hij^
5:1	25.06 ± 2.44^eg^	10.13 ± 1.25^eg^	663.59 ± 89.99^ab^	50.78 ± 6.69^eg^
7:1	24.39 ± 2.83^eg^	10.00 ± 2.00^eg^	633.02 ± 77.63^ab^	50.30 ± 6.14^eg^
9:1	23.79 ± 2.56^eg^	10.63 ± 2.67^eg^	600.25 ± 96.45^ab^	48.45 ± 4.79^eg^

*OVX* Bilateral ovariectomy, *RES* Risedronate sodium, *EAP* Exopolymers purified from *A. pullulans* SM2001, *TM T. morbifera* leaf extracts, *(EAP:TM)* Mixed formula consisted of EAP and TM (*g*/*g*), *TV/BV* Trabecular bone volume (%), *Tbn* Trabecular bone number (Numbers/epiphyseal), *Tbl* Trabecular bone length (Longitudinal thickness; μm), *Tbt* Trabecular bone thickness (Cross thickness; μm)

Values are expressed mean ± S.D. of eight mice

^a^*p* < 0.01 as compared with sham control by LSD test

^b^*p* < 0.01 as compared with OVX control by LSD test

^c^*p* < 0.01 as compared with EAP single formula treated mice by LSD test

^d^*p* < 0.01 as compared with TM single formula treated mice by LSD test

^e^*p* < 0.01 and ^f^*p* < 0.05 as compared with sham control by MW test

^g^*p* < 0.01 and ^h^*p* < 0.05 as compared with OVX control by MW test

^i^*p* < 0.01 as compared with EAP single formula treated mice by MW test

^j^*p* < 0.01 as compared with TM single formula treated mice by MW test

**Table 5:** Histopathology and histomorphometry of the femur in sham-operated and OVX mice: Cortical Bones and Osteoclast Cells

Items Groups	Left femur tissues		
	Cbt	Ocn	OS/BS
Controls			
Sham	305.93 ± 46.58	3.00 ± 1.31	3.70 ± 1.94
OVX	171.11 ± 17.15^a^	19.63 ± 2.50^a^	25.29 ± 6.10^a^
Reference (2.5 mg/kg)			
RES	170.51 ± 11.52^a^	20.25 ± 4.53^a^	10.09 ± 1.86^ab^
Single formula (200 mg/kg)			
EAP	196.82 ± 12.34^ab^	13.88 ± 2.36^ab^	15.53 ± 2.37^ab^
TM	188.84 ± 9.85^ac^	13.13 ± 2.10^ab^	14.48 ± 3.30^ab^
Mixed formula - EAP:TM (200 mg/kg)			
1:1	195.02 ± 9.43^ab^	13.50 ± 1.77^ab^	15.20 ± 1.71^ab^
1:3	191.78 ± 9.42^ac^	13.25 ± 3.15^ab^	14.79 ± 4.39^ab^
1:5	189.98 ± 8.23^ac^	13.75 ± 1.91^ab^	16.18 ± 1.78^ab^
1:7	190.90 ± 7.34^ac^	12.50 ± 2.45^ab^	14.86 ± 2.38^ab^
1:9	190.56 ± 10.85^ac^	13.13 ± 1.73^ab^	14.93 ± 2.49^ab^
3:1	236.15 ± 33.30^abde^	6.63 ± 1.41^abde^	7.18 ± 1.41^abde^
5:1	194.38 ± 10.32^ab^	12.50 ± 1.60^ab^	13.91 ± 2.35^ab^
7:1	194.87 ± 10.65^ab^	12.25 ± 2.71^ab^	14.02 ± 3.30^ab^
9:1	196.31 ± 10.93^ab^	13.25 ± 2.05^ab^	15.14 ± 1.73^ab^

*OVX* Bilateral ovariectomy, *RES* Risedronate sodium, *EAP* Exopolymers purified from *A. pullulans* SM2001, *TM T. morbifera* leaf extracts, *(EAP:TM)* Mixed formula consisted of EAP and TM (*g*/*g*), *Cbt* Cortical bone thickness (Cross thickness; μm), *Ocn* Osteoclast cell number (Numbers/epiphyseal), *OS/BS* Osteoclast cell surface/bone surface (%)

Values are expressed mean ± S.D. of eight mice

^a^*p* < 0.01 as compared with sham control by MW test

^b^*p* < 0.01 and ^c^*p* < 0.05 as compared with OVX control by MW test

^d^*p* < 0.01 as compared with EAP single formula treated mice by MW test

^e^*p* < 0.01 as compared with TM single formula treated mice by MW test

#### Bone structure and mass

The OVX controls displayed significant (*p < 0.01*) decreases in femur Tbl, Tbn, Tbt, Cbt and TV/BV as compared to the sham-operated controls. However, all EAP:TM formulations including the single formulations significantly (*p < 0.01; p < 0.05*) inhibited the decreases in bone structure and mass. In particular, EAP:TM (3:1)-treated mice showed significantly (*p < 0.01*) increased inhibition of bone mass depletion and destruction of structures induced by OVX. RES treated groups also displayed increases in TV/BV, Tbn, and Tbl of the left femur, but femur Cbt and Tbt showed no significant differences compared with that of OVX mice (Tables 4 and 5; Fig. 5; Additional file 7).

#### Bone resorption

OVX control mice displayed increased femur Ocn and OS/BS ratios as compared with the sham control mice. However, the stimulation and escalation of osteoclast cells was significantly (*p < 0.01*) repressed by treatment with all EAP:TM formulations including the single formulations; particularly, EAP:TM 3:1-treated groups consistently exhibited significant (*p < 0.01*) increases in the inhibition of osteoclast cell activation and proliferation. Although RES-treated mice exhibited comparable left femur Ocn as that of the OVX control mice, OS/BS ratios were significantly deceased as compared to that in the OVX controls (Table 5; Fig. 5; Additional file 8).

## Discussion

Estrogen deficiency during menopause is one of the major causes of osteoporosis in menopausal women [54]. A disturbance in bone formation and resorption results in fractures and bone loss, ultimately causing osteoporosis [2]. Antiresorptive agents are well known compared to the bone formation stimulant anabolic agents [6]. In depth understanding of bone formation and osteoblast differentiation has helped in the design of trials to develop anabolic agents [7].

*T. morbifera* has traditionally been used as an ingredient in preparing medicine and tea for various pharmacological purposes [16–18]. The bone-preserving properties of exopolysaccharide (EAP) have been potentiated by combining it with other active agents [47–49]. Therefore, we expected that appropriate combinations of EAP and TM would demonstrate promising synergistic anti-osteoporotic activities due to the increased availability of the bioactive substances. During this experiment, we used bilateral OVX mice, a renowned rodent model for studying human osteoporosis [7–9], to observe the synergistic anti-osteoporotic potential of mixtures containing different proportions of EAP and TM compared with that of the single formulation of each herbal extract. The EAP:TM mixtures in the present study, significantly (*p* < 0.01 or *p* < 0.05) increased anti-osteoporotic activity compared to single formulations of EAP or TM, providing evidence for the synergistic effects of combining these two herbal extracts.

OVX mice showed noticeable increases in body weight, weight gain, and serum osteocalcin levels, as well as decreases in serum bALP activity, femur wet, dry, and ash weights, femur Ca and IP contents, BMD, and strength as compared to sham vehical control. In addition, decreases in all histomorphometric indices indicated reduction in bone mass and structure, and increases in resorption indices in the femur of OVX controls suggested increases in bone turnover and decreases in bone formation, which are associated with estrogen-deficient osteoporosis. However, these OVX-induced estrogen-deficient osteoporotic signs relating to decreases in bone formation and increases in bone turnover were considerably inhibited by 35 days of continuous oral application with single formulations of EAP or TM, as well as all 9 mixtures of EAP:TM, particularly, EAP:TM (3:1) formulation. These outcomes of this study advocate that the EAP:TM (3:1) mixture synergistically enhanced the distinctive anti-osteoporotic effects of the single formulations, likely due to the increased availability of the bioactive substances.

The results of the present study were comparable to those of RES (2.5 mg/kg) treatment in this experiment, which suggest that the EAP:TM formulation 3:1 might act as a potent new agent for curing osteoporosis in menopausal women. Although RES 2.5 also ameliorated decreases in femur BMD, femur strength, and trabecular bone architectures induced by estrogen-deficient OVX (through inhibition of bone turnover), it did not critically affect bone formation. The observed serum bALP activity and cortical and trabecular bone thickness were consistent with previously reported results [14, 15]. In one condition of the current experiment, EAP:TM 3:1 showed slightly lower antiresorptive effects compared to RES (2.5 mg/kg). However, EAP:TM 3:1 exhibited effects on bone formation, which were not observed with RES 2.5 mg/kg treatment. Hence, EAP:TM 3:1 demonstrated favorable anti-osteoporotic effects in OVX mice that were comparable to RES (2.5 mg/kg) treatment.

During the present study, the observed increases in weight gain and body weights in the OVX groups were distinct signs of estrogen-deficient condition in the tested mice, however the differences were inconsistent. Lorden and Caudle (1986) as well as Bain et al. (1993) reported increases in body weight after OVX [55, 56], while other studies failed to report the same [57, 58]. Generally, decreases in body weight or weight gain have been considered a sign of toxicity in normal states but in disease states, such as obesity, it is regarded as a favorable sign [7, 9]. During this study, noteworthy decreases in bodyweight gains were observed in all mice treated with EAP:TM formulations, particularly in EAP:TM 3:1-formulation.

Changes in bone weight are not considered a significant parameter for detecting the efficacy of an anti-osteoporotic agent [59]. However, increases in relative bone weights have been deliberated as a valued indicator of anti-osteoporotic property [7, 9]. In the present study, OVX mice showed noteworthy decreases in femur absolute and relative dry and ash weights, as well as relative wet-weights. Conversely, marked enhancements in femur dry, wet, and ash weights were noticed in mice treated with all test materials, including RES (2.5 mg/kg). In particular, EAP:TM 3:1-formulation displayed significant increase in femur absolute and relative ash and dry weights, as well as wet-weights. Hence, the inhibitory effects of TM or EAP single formulations on OVX-induced decreases of bone weights were synergistically enhanced by the EAP:TM 3:1 combination.

Variations in the use of animals, and study design have been reported by different studies. There also exist disagreements on the interpretation of bone turnover and formation. Despite these issues, serum bALP and osteocalcin levels are normally considered as acceptable indicators of bone formation and turnover, respectively [7, 9, 60–62]. In this study, OVX-control groups showed a significant decrease in serum bALP activity and a significant increase in serum osteocalcin levels as compared with the sham-control groups. However, all EAP:TM single or mixed formulations and especially, mice treated with EAP:TM 3:1-formualtion showed increases in serum bALP activity and decreases in osteocalcin levels.

Bone mineral content significantly decrease as osteoporosis progresses [7, 9]. Specifically, IP and Ca decrease most dramatically; however, Ca/IP ratios do not change because of the simultaneous reduction in both IP and Ca concentration [7, 9, 63, 64]. In the current analysis, significant decreases in femur Ca and IP concentrations were observed in OVX control. Nevertheless, marked increases in femur Ca and IP levels were noticed in all mice administered the test materials, including EAP:TM 1:1, compared with that in OVX control mice. In particular, EAP:TM 3:1-treated mice also revealed marked increases in Ca and IP contents in the right femur.

BMD, a well-acknowledged indicator of fluctuations in bone quality under clinical settings, decreases in osteoporosis, irrespective of the cause [7, 9, 65, 66]. BMD has provided useful predictive information on the efficacy of anti-osteoporotic agents [66], and bone quality diagnostic profiles for clinical research [65]. BMD and bone strength significantly reduces in osteoporosis irrespective of the causes [7, 9, 65, 66]. In this study, a significant decrease in femur mean BMD and total body was noticed in OVX control groups. However, test material-administered groups including those treated with the EAP single formulation, showed noteworthy increases in femur FL and mean BMD, and total body. Especially, EAP:TM 3:1 (g/g)-treated mice showed significant enhancement in femur mean BMD, total body and femur strength as compared to those of EAP or TM single formulations.

The changes in bone morphology can be easily observed by microscopic analysis [7, 9, 67]. The histological profiles of cortical and trabecular bones mainly alter in osteoporotic animals. Furthermore, the impact of numerous anti-osteoporotic substances has been estimated on bone histology [68]. Some histomorphometric indices of bone masses noticeably decrease whereas, bone resorption indices increase; this kind of measurements have been found to be good predictors of the efficacy of anti-osteoporotic agents [9, 68–70]. In this study, OVX-control groups showed typical osteoporotic histological profiles, as evidenced by dramatic reduction in cortical and trabecular bone mass, as well as enhancement in connective tissue of the cortical bone. Although administration of RES repressed trabecular bone loss, it did not affect cortical bone mass. Whereas, significant enhancement in the cortical and trabecular bone structure and mass was observed in all groups administered with the test materials including single formulations. This could be attributed to the inhibition of osteoclast cell activity by EAP and TM. In particular, EAP:TM 3:1-treated mice showed greater inhibition of the histopathological reduction in osteoclast activation and femur bone mass compared to mice administered with the TM or EAP single formulations.

The findings of the present study clearly indicated the favorable synergistic effects of EAP:TM (3:1) formulation on bone turnover and formation in OVX-induced metabolic disorder. No remarkable variation in body weight, body weight gain, and femur Ca/IP ratio was detected in OVX control groups as well as in groups administered with the test materials, including RES. Moreover, RES 2.5 mg/kg significantly decreased serum osteocalcin levels, but had no effect on serum bALP activity.

## Conclusions

In this study, a continuous oral administration of the single and/or combined formulations of EAP and TM significantly inhibited the OVX-induced osteoporotic symptoms i.e., decreased formation and increased turnover of bone. In particular, compared with the single formulations, the EAP:TM (3:1) mixture revealed significantly favorable inhibition of estrogen-deficient osteoporosis indices. In the conditions of the current experiment, EAP:TM (3:1) formulation showed slightly lower antiresorptive effects than RES 2.5 mg/kg; however, EAP:TM 3:1 demonstrated bone formation effects, which were not observed with RES 2.5 mg/kg treatment. These findings are considered direct and reliable proof that the EAP:TM 3:1 mixed formulation synergistically enhanced the distinct anti-osteoporotic potential of the single EAP or TM formulations, which might be due to the enhanced availability of the bioactive substances. Hence, EAP:TM 3:1 mixture might act as a potent new agent for curing osteoporosis in menopausal women. Although RES 2.5 mg/kg also favorably ameliorated OVX-induced reductions in femur BMD, femur strength, and trabecular bone architecture through the inhibition of bone turnover, it did not critically affect bone formation (measured by serum bALP and cortical and trabecular bone thickness).

## Additional files

Additional file 1: Raw data on body weight gain in sham-operated and OVX mice. OVX: Bilateral ovariectomy; RES: Risedronate sodium; EAP: Exopolymers purified from *A. pullulans* SM2001; TM: *T. morbifera* leaf extracts; (EAP:TM): Mixed formula consisted of EAP and TM (*g*/*g*). Values are expressed mean ± S.D. of eight mice. *All animals were overnight fasted. (XLSX 12 kb)

Additional file 2: Raw data on right femur weights of sham-operated and OVX mice. OVX: Bilateral ovariectomy; RES: Risedronate sodium; EAP: Exopolymers purified from *A. pullulans* SM2001; TM: *T. morbifera* leaf extracts; (EAP:TM): Mixed formula consisted of EAP and TM (*g*/*g*). Values are expressed mean ± S.D. of eight mice. (XLSX 32 kb)

Additional file 3: Raw data on serum osteocalcin and bALP levels in sham-operated and OVX mice. OVX: Bilateral ovariectomy; RES: Risedronate sodium; EAP: Exopolymers purified from *A. pullulans* SM2001; TM: *T. morbifera* leaf extracts; (EAP:TM): Mixed formula consisted of EAP and TM (*g*/*g*). Values are expressed mean ± S.D. of eight mice. RES was orally administered at a dose level of 2.5 mg/kg. All single and mixed formula consisted of EAP and TM were administered at a dose level of totalized 200 mg/kg by gastric gavage. (XLSX 15 kb)

Additional file 4: Raw data on BMD (bone mineral density; total body and right femur). OVX: Bilateral ovariectomy; RES: Risedronate sodium; EAP: Exopolymers purified from *A. pullulans* SM2001; TM: *T. morbifera* leaf extracts; (EAP:TM): Mixed formula consisted of EAP and TM (*g*/*g*). Values are expressed mean ± S.D. of eight mice. (XLSX 15 kb)

Additional file 5: Raw data on FL (failure load) quantified bone strength. OVX: Bilateral ovariectomy; RES: Risedronate sodium; EAP: Exopolymers purified from *A. pullulans* SM2001; TM: *T. morbifera* leaf extracts; (EAP:TM): Mixed formula consisted of EAP and TM (*g*/*g*). Values are expressed mean ± S.D. of eight mice. (XLSX 13 kb)

Additional file 6: Raw data on BMC (bone mineral content), Ca and IP. OVX: Bilateral ovariectomy; RES: Risedronate sodium; EAP: Exopolymers purified from *A. pullulans* SM2001; TM: *T. morbifera* leaf extracts; (EAP:TM): Mixed formula consisted of EAP and TM (*g*/*g*). Values are expressed mean ± S.D. of eight mice. Ca = Calcium; IP = Inorganic phosphorus. (XLSX 19 kb)

Additional file 7: Raw data for Histopathology and Histomorphometry of the femur in sham-operated and OVX mice: Trabecular Bones. OVX: Bilateral ovariectomy; RES: Risedronate sodium; EAP: Exopolymers purified from *A. pullulans* SM2001; TM: *T. morbifera* leaf extracts; (EAP:TM): Mixed formula consisted of EAP and TM (*g*/*g*). Values are expressed mean ± S.D. of eight mice. TV/BV = Trabecular bone volume (%); Tbn = Trabecular bone number (Numbers/epiphyseal); Tbl = Trabecular bone length (Longitudinal thickness; μm); Tbt = Trabecular bone thickness (Cross thickness; μm). (XLSX 23 kb)

Additional file 8: Raw data for Histopathology and Histomorphometry of the femur in sham operated and OVX mice: Cortical Bones and Osteoclast Cells. OVX: Bilateral ovariectomy; RES: Risedronate sodium; EAP: Exopolymers purified from *A. pullulans* SM2001; TM: *T. morbifera* leaf extracts; (EAP:TM): Mixed formula consisted of EAP and TM (*g*/*g*). Values are expressed mean ± S.D. of eight mice. Cbt = Cortical bone thickness (Cross thickness; μm); Ocn = Osteoclast cell number (Numbers/epiphyseal); OS/BS = Osteoclast cell surface/bone surface (%). (XLSX 15 kb)

## Abbreviations

ANOVA: One way analysis of variance
bALP: Bone specific alkaline phosphatase
BMD: Bone mineral density
Ca: Calcium
Cbt: Cortical bone thickness
EAP: Extracellular polymers isolated from *Aureobasidium pullulans* SM-2001
FL: Failure load
HE: Hematoxylin and eosin
ICR: Institute of Cancer Research
IP: Inorganic phosphorus
LSD: Least-significant differences
MW: Mann-Whitney U
Ocn: Osteoclast cell number
RES: Risedronate sodium
Tbl: Trabecular bone length
Tbn: Trabecular bone number
Tbt: Trabecular bone thickness
TBV: Trabecular bone volume
TM: *Textoria morbifera*
TV/BV: Trabecular bone volume (%)
